# Recruitment of scarce competences to rural regions: Policy perspectives

**DOI:** 10.1007/s10037-021-00155-w

**Published:** 2021-09-15

**Authors:** Kristina Nyström

**Affiliations:** 1 Division of Sustainability, Industrial Dynamics & Entrepreneurship, Department of Industrial Economics and Management, KTH, The Royal Institute of Technology, Lindstedsv 30, SE‑100 44 Stockholm, Sweden; 2 The Ratio Institute, P.O Box 3203, SE103 64 Stockholm, Sweden

**Keywords:** Recruitment, Skills matching, Rural development, Regional policy, R23, R28

## Abstract

This paper studies the perceived difficulty of recruiting scarce competencies to rural regions. Furthermore, the role of policy in facilitating and enhancing recruitment to and better skills matching in rural regions is discussed. Based on a survey targeted to the business sections of Swedish municipalities, the results show that recruitment is perceived to be difficult in both rural and nonrural regions and that the difficulty of recruiting for the right skills results in a lack of skills matching and constitutes an obstacle to growth. Rural regions located close to urban areas can to some extent mitigate these recruitment problems, and their locations pose less of a barrier in recruitment processes compared to those of remotely located rural regions.

Which policies can help remedy recruitment problems faced in rural regions? In both rural and nonrural regions, incentives for writing off student debt and relocation support for accompanying persons and tandem recruitment are perceived to be the most promising policies. Rural regions are more receptive to the implementation of such policies. Finally, the need for flexibility and policies that can be adapted to the regional demand for labour are stressed.

## Introduction

Successful recruitment is vital to businesses’ ability to adapt and develop and hence for their potential growth and long-term survival. Nevertheless, many employers encounter problems with recruiting employees, which constitutes an obstacle to the growth or even the survival of their ventures. In the Swedish context, approximately 1/3 of businesses perceive access to suitable labour and skills as an obstacle to growth, and this obstacle is regarded as a more important obstacle to growth than, for example, rules and regulations or access to finance (Swedish Agency for Economic and Regional Growth 2020a).

In addition to being an obstacle to firm growth, recruitment problems may lead to skill mismatches. According to the OECD (2016), approximately 40% of Swedish employees are mismatched in terms of qualifications, while approximately 10% are mismatched in terms of skills. At the aggregate level, skill mismatch implies a less efficient allocation of resources, and hence, skill mismatch will have negative effects on labour productivity (Adalet McGowan and Andrews 2015). Furthermore, skill mismatch is shown to have negative effects on, for example, earnings, job satisfaction and human capital accumulation (see, e.g., Mavromaras et al. 2013). Overall, recruitment problems and skill mismatches are identified as some of the main challenges facing the Swedish economy and have the potential to negatively influence economic growth (see, e.g., World Bank 2014).

Recruitment problems and skill mismatches are expected to be particularly pronounced in small and remote rural regions. For many job applicants, peripheral areas are perceived to be less attractive than metropolitan areas and urban agglomerations (Buenstorf et al. 2018). According to the previous literature, workers in rural regions face a number of issues, including a lack of professional job opportunities; limited opportunities to gain and broaden their work experience and problems with accessibility, such as limited transport and mobility (De Hoyos and Green 2011). However, the problems that employers in rural regions face have received less attention in the literature (De Hoyos and Green 2011).

The successful attraction and retention of employees in rural regions calls for thinking outside the box from the employer’s perspective.

Are you someone who is looking to live a simpler life, close to nature, in an area that still believes in community meals and weekly jam sessions? We can’t give you big money, but we can give you an awesome life. (CBC News 2016a).

A small family-owned business in the small village of Whycocomagh in Nova Scotia, Canada tried to recruit employees to this beautiful but less populated region using the above arguments in a Facebook ad. In addition to securing a job, employees willing to relocate would receive two acres of land (CBC News 2016a). This serves as one example of how employers use innovative initiatives to recruit employees to rural regions. Recruitment problems in rural regions have also been acknowledged by policymakers at both the regional and government levels. Policy efforts aimed at strengthening competencies are often appreciated and serve as a lever for company development (Swedish Agency for Economic and Regional Growth 2016). However, as emphasized even by Swedish government authorities, knowledge of scarce competencies and recruitment in a regional context remains limited (Swedish Agency for Economic and Regional Growth 2020b).

This paper aims to contribute to knowledge on the recruitment of scarce competencies to rural regions. In the discussion of skills shortages in rural areas, attention has often been paid to recruitment problems and scarce competencies in the public sector, such as shortages of nurses and physicians (see, e.g., Jones et al. 2019), and the WHO (2010) has raised concerns about health worker shortages in rural areas of high-income countries. However, less attention has been given to such shortages in the business sector. Accordingly, to fill this research gap, this paper examines perceived shortages of scarce competencies and recruitment problems faced in both the public and business sectors. Are there differences in the severity of recruitment problems found in rural and nonrural municipalities?

Employers’ recruitment to and retention of employees in rural areas cannot be studied in isolation. It is crucial to also consider demographic, economic, and political contexts (De Hoyos and Green 2011). Hence, this paper also aims to address policy perspectives and to discuss policies that can enhance the recruitment of scarce competencies. Which policies are perceived to be plausible and effective in enhancing the recruitment of scarce competencies to rural regions? The study’s empirical context focuses on a survey of representatives from 290 Swedish municipalities, 130 of which are defined as rural.

This paper is organized into five sections. Sect. 2 discusses the theoretical arguments and previous empirical research regarding recruitment to rural regions. Furthermore, an overview of policy measures aimed at enhancing recruitment and retention to rural regions and of how they have been evaluated is provided. Section three describes the empirical and data collection approaches adopted. The empirical results are provided in section five. Finally, section five provides a concluding discussion.

## Recruitment of employees to rural regions

Employee recruitment necessitates a matching process whereby both the employee and employer must find a satisfactory match. It can be argued that the recruitment process and skills matching are particularly difficult for firms located in small and rural regions. In this section, the challenges and opportunities facing rural regions are discussed. Furthermore, evidence of the effectiveness of policies is surveyed.

### Labour market challenges and opportunities for rural regions

Location decisions are important for both entrepreneurs and individuals. For an entrepreneur starting and running a business, several factors need to be considered. Access to local demand, the supply of input factors, agglomeration economies, and the policy environment are some of such factors (see, e.g., Johnson and Parker 1996 and Nyström 2007). Regarding local demand, the number of potential customers and their incomes may be relevant decision-making factors. The supply of input factors may be premised on proximity to natural resources but is also based on knowledge and skills. The benefits of an urban location are related to the benefits of *sharing, matching* and* learning* in agglomerated regions (Duranton and Puga 2004). In agglomerated regions, indivisible goods and facilities can be s*hared,* and firms and individuals can benefit from sharing the variety of goods and services provided. In larger regions, the number of employers and employees looking for a match is larger. Accordingly, both the probability and quality of* matching* increase. Finally, location in agglomerated areas increases the possibility of knowledge spillovers, which is beneficial to *learning* and innovation (Duranton and Puga 2004). Forces of access to local demand, access to specialized knowledge and benefits of agglomeration economies in terms of better matching and learning constitute a centripetal force (Krugman 1999), making dense regions an attractive location decision.

However, locating close to other firms may also have a negative effect, such as increased wages and increased input prices and congestion with respect to, for example, traffic and housing. An important mechanism of diseconomies of agglomeration is higher land prices. These diseconomies of scale contribute centrifugal forces, making rural regions relatively more attractive (Krugman 1999).

In applying the above outlined theoretical arguments on the opportunities and challenges of less agglomerated areas more explicitly to rural regions, it is argued that the existing pool of potential employees is small (when a firm recruits locally) in these regions. Hence, the potential to find perfect matches among hiring demands, employee competencies and employee relocation ability/desires may be more difficult to achieve in small and rural regions. The size of a region is of utmost importance in successful labour market matching. For instance, employees who suffer from displacements are more likely to be reemployed in metropolitan regions (Nyström 2018). By extending their recruitment area, employers can naturally extend the pool of potential applicants (perhaps even recruiting globally), but they may also then face issues related to attracting (retaining) employees who are willing to move to a rural region. In addition, issues related to finding employment for spouses and attractive opportunities for accompanying family members may also be crucial to relocation decisions. The possibility of extending the recruitment area increases somewhat when a rural area is located close to urban areas.

With regard to the mobility of highly qualified workers, a body of literature examines the motives and behaviours of, for instance, recent graduates and the influence of regional economic conditions, i.e., the overall supply of employees, on their mobility (Winterhager and Krücken 2015). However, according to Winterhager and Krücken (2015), this literature does not consider the demand side of labour markets. In addition, there is a lack of literature that considers regional aspects of the recruitment processes of highly qualified workers (Winterhager and Krücken 2015). When regional aspects are considered, they are seen as a subfactor of contextual characteristics or as organizational characteristics (Rynes and Cable 2003; Uggerslev et al. 2012). Hence, there is a gap in our knowledge of the recruitment of highly skilled workers from a regional perspective.

What drives individuals’ decisions to relocate to rural areas and remain there? For simplicity, we distinguish between pecuniary and nonpecuniary incentives. Financial incentives may be of less importance than may be expected. In fact, empirical evidence shows that a substantial proportion of job changes are associated with lower wages (see, e.g., Jolivet et al. 2006). For Sweden, Nyström and Zhetibaeva Elvung (2015) find that approximately 30% of voluntary job changes come with a wage decrease. In further showing that factors other than wages are important in employment decisions, Terjesen et al. (2007) find that organizational attributes, such as investment in training and development and opportunities for long-term career progression, are more important than a high starting wage. According to Gordon (2015), an important factor for occupational advancement is exposure to superior learning opportunities and, in particular, tacit knowledge. Where does one find this knowledge? According to Gordon (2015), one most likely finds such knowledge close to cutting-edge markets or technology changes where the customization of products/services for quality-sensitive clients is performed and in locations with sophisticated forms of collaboration. These places are likely to be concentrated to a limited set of high-order centres/city regions.

Applying a regional perspective, Yang (2003) surveyed urban physicians and found that 70% would, under no circumstances, consider moving to a rural area, and those who would consider this would expect a substantial wage increase (approximately 35%). Instead, nonpecuniary factors are of great importance. Factors frequently cited to influence these decisions include job opportunities for partners, perceptions of rural life, housing and health provision, place identity and social networks, education and training, and infrastructure (Bureau of Transport Regional Economics 2006).

Among the nonpecuniary aspects involved in the retention of physicians in rural areas, Mathews et al. (2012) also stress the importance of the work environment and of organizational culture. Furthermore, the influence and support of partners is identified as a key factor in the recruitment to and retention of employees in rural areas (e.g., Mayo and Mathews 2006). Regarding the importance of access to education, Yang (2003) found that both physicians in rural areas and those in urban areas perceive the quality of children’s education to be lower in rural/remote areas.

Bjerke and Mellander (2017) find that family structure is a strong driver of location decisions. Their study of mobility patterns for individuals after finalizing university education shows that university graduates with children are more likely to move back to their home regions. Regarding mobility to rural regions that are not their home regions, singles with no children are more likely to locate to such regions after graduation. However, such individuals are more likely to move away later on when they have children.

### Policy measures targeted towards recruitment to rural areas

As mentioned above, this paper provides a discussion of policies that can support the recruitment of employees to rural regions. This discussion includes examples from Norway and Finland, which suffer from similar recruitment problems in rural areas of northern regions. What are the experiences of countries that have implemented policy support in terms of, for instance, lowered tax deductions and wage subsidies to incentivize relocation to remote rural regions?^1^ In the following discussion, we distinguish between policies using pecuniary tools at either the firm or individual level and nonpecuniary incentives, which aim at, for example, reducing information asymmetries and enhancing the functioning of the local labour market.

#### Pecuniary measures targeted towards firms/organizations

##### Differentiated social security contributions

Norway has a long history of implementing differentiated social security contributions at the regional level (since 1975). Norway is divided into 7 zones with 0–14.1% social security contributions. Applying differentiated social security of state aid requires that for each period the setup be negotiated and accepted by the European Free Trade Association (EFTA), of which Norway is one of the four members. According to an evaluation by Angell et al. (2012), the effect of lower social security fees varies across industries. Manufacturing tends to transfer lower payments to higher wages or profits, while an employment effect is visible in the service industry. However, in the longer term (five years), indirect employment effects are found, which is in line with other studies on differentiated social security contributions (Angell et al. 2012).

The Swedish Agency for Economic and Regional Growth (2010) provides an overview of empirical research on the effects of regionally differentiated social security fees at the firm level. Available studies tend to focus on employment and wage effects, and the time horizon is short in most cases. A review of these studies provides limited support for any employment effects. For instance, Korkeamäki and Uusitalo (2009) study the impact of reduced payroll taxes (3–6%) in northern Finland and find that wages increased but with no effect on employment. Bennmarker et al. (2009) investigate the effects of a social security fee reduction of ten percentage points introduced in northern Sweden in 2002 and do not find any employment effects on existing firms. However, the authors do observe that the wages in these companies increased by 0.25% with each percentage point of tax reduction and find a slightly positive effect of the number of firms and hence a slightly positive employment effect overall.

##### Wage subsidies

In 1990, a policy package called the “Action Zone for Finnmark County and Nord-Troms Region” was introduced in northern Norway. One part of the policy package involved wage subsidies for preschool teachers. However, this policy was repealed in 2012 (Angell et al. 2016) and evaluated together with the rest of the regional policy package (see the findings below).

#### Pecuniary measures targeted towards individuals and families

##### Tax reductions for individuals

The previously mentioned Action Zone policy package adopted in Norway also included tax reductions for individuals. The tax reductions had differing components, resulting in the personal tax rate in Finnmark and Nord Troms being 3.5 percentage points lower than that for the rest of the country (Angell et al. 2016).

##### Student debt relief

Another part of the Action Zone policy package involved the introduction of student debt relief, where individuals with loans from the Norwegian State Educational Loan Fund could obtain debt relief of 10% per year. In the Swedish context, a recent government committee suggested that the government investigate the possibility of reducing student loans in 23 specifically targeted municipalities (SOU 2017). The proposed model is similar to the Norwegian model with a 10% annual reduction and a maximum reduction of approximately EURO 3,000 per year.

##### Family allowance

As part of the Action Zone policy package, the family allowance was increased by approximately EURO 300 per year and child. This policy was repealed in 2014 (Angell et al. 2016).

An evaluation of the Action Zone policy package showed that the number of people living in the region was stabilized, particularly for the most mobile groups, which include individuals without any previous personal connection to the region where they live. However, highly educated individuals are still highly mobile. It was also concluded that the introduction of personal incentive structures increased the probability of remaining in the region and stimulated recruitment to the region (Agnell et al. 2012).

#### Nonpecuniary initiatives

As mentioned above, Yang (2003) provides evidence that pecuniary incentives are perceived to be less important in many decisions to relocate to a rural area. From a policy perspective, Yang (2003) argues that this suggests that alternatives to financial incentives need to be explored. However, such policies seem to be less prevalent and less commonly evaluated, at least according to the research literature. Potential areas that may be of interest include nonpecuniary support in finding proper housing and measures aimed at supporting family relocation. There are also examples of municipalities that help organize recruitment trips to countries and locations where candidates interested in relocation may be found.

To summarize the evidence regarding the policy measures discussed in this section, it can be concluded that there seems to be some evidence of positive effects of pecuniary support at the individual level. However, at the firm level, there is limited empirical evidence in support of employment effects. However, nonpecuniary measures have been less explored, and we lack systematic knowledge of whether and how they are used at the regional level.

## Data and method

A questionnaire aimed at studying skills shortages, recruitment, and perceptions of related policies was conducted in the spring of 2020. The questions included both multiple-choice and open-ended questions to deepen the possibility of obtaining insights into the topic. For the multiple-choice questions, respondents were asked about the extent to which they agreed with a number of statements. A Likert scale with seven alternatives ranging from measures “do not agree at all” to “agree completely” was used. For data compilation, these answers were coded as choices 1–7, where “does not agree at all” corresponded to a value of 1 and “agree completely” corresponded to a value of 7. In the analysis of the results, the mean value and standard deviation were determined. Furthermore, a t-test was employed to determine any statistically significant differences between respondents from rural and nonrural municipalities. In addition, t‑tests checked for possible statistically significant differences between the two types of rural regions, i.e., rural municipalities located close to urban regions and rural and remote rural municipalities. For some parts of the analysis, it is interesting to note the proportion of respondents who agreed or disagreed with each statement. When a respondent chose an answer corresponding to a value of 1, 2 or 3, the respondent was assumed to not agree with the statement. Those selecting an answer corresponding to a value of 5, 6 or 7 were assumed to agree with the given statement to some extent.^2^

The questionnaire was distributed to the heads of the business sections^3^ (or equivalent function) of each of the 290 Swedish municipalities. We selected municipality officials as targets for the questionnaire because they are in constant dialogue with business organizations and entrepreneurs in their regions. Furthermore, such individuals sometimes commission or conduct their own investigations on labour shortages in their region. Hence, we expected them to have good knowledge about recruitment problems and of which policy measures may help circumvent such problems in their particular regions. The contacted persons were identified with the help of the municipalities’ websites.^4^ The survey was active from the end of February to mid-April 2020. During the month of March, Sweden began feeling the effects of the COVID-19 pandemic. This likely affected the response rate of the latter part of the survey, as the majority of respondents stated that they needed to prioritize providing support to the municipality’s companies given the prevailing circumstances. When the survey was closed, representatives from 110 municipalities completed the survey, corresponding to a response rate of 38%.

Since the purpose of the survey was to identify possible differences between rural and nonrural municipalities, the compilation of results from municipalities defined by the Swedish Agency for Growth Policy Analysis (2014) as rural (130 municipalities) and nonrural municipalities was used to define rural versus nonrural regions. Since accessibility to urban regions may be important for the recruitment possibilities of rural regions, the distinction between rural municipalities in remote locations and rural municipalities located close to urban regions is also used. Seventy municipalities are defined as rural areas located close to urban regions, and 60 municipalities are defined as rural areas in remote locations.^5^ For the rural municipalities, representatives from 49 municipalities participated in the survey, corresponding to a response rate of 38%, which is the same as the overall response rate.

## Results

Table 1 provides a summary of the results regarding perceptions of recruitment problems and consequences for the given regions. How severe are recruitment problems in rural regions? The general findings suggest that recruitment is challenging in all regions and in both the public and business sectors since the Likert scale measurement shows quite high values. However, a comparison of recruitment to the public and business sectors shows that difficulties are somewhat more pronounced in the business sector.^6^

**Table 1:** Recruitment and scarce competences from a regional perspective

	Mean	St. dv.	Difference (mean)
**To what extent do you agree with the following statements?**			
*It is difficult to recruit staff for the municipality’s public activities*			
Nonrural municipalities	4.604	1.364	–
Rural municipalities	4.958	1.166	0.354
Rural and located close to urban region	4.545	1.184	–
Rural and remote location	5.308	1.050	0.763^##^
*It is difficult for businesses in the municipality to recruit staff*			
Nonrural municipalities	5.050	1.204	–
Rural municipalities	5.184	0.993	0.134
Rural and located close to urban region	5.091	1.065	–
Rural and remote location	5.259	0.944	0.168
*The difficulties in recruiting result in a lack of skills matching*			
Nonrural municipalities	5.556	1.369	–
Rural municipalities	5.510	1.277	−0.046
Rural and located close to urban region	5.545	1.405	–
Rural and remote location	5.481	1.189	−0.064
*The difficulty of recruiting the right skills is an obstacle to growth in our municipality*			
Nonrural municipalities	5.759	1.329	–
Rural municipalities	5.653	1.332	−0.106
Rural and located close to urban region	5.500	1.504	–
Rural and remote location	5.778	1.188	0.278
*The municipality’s geographical location is a disadvantage in terms of recruitment*			
Nonrural municipalities	3.167	1.932	–
Rural municipalities	4.354	2.109	1.187***
Rural and located close to urban region	3.286	2.148	–
Rural and remote location	5.185	1.688	1.899^###^

* indicates a statistically significant difference between rural and non-rural municipalities. *p < 0.10, **p < 0.05, ***p < 0.01

# indicates a statistically significant difference between rural and located close to urban region and rural and remote locations. ^#^*p* < 0.10, ^##^*p* < 0.05, ^###^*p* < 0.01

A comparison of the results for rural and nonrural regions does not indicate any statistically significant differences between the public and business sectors. However, a closer examination of the two types of rural regions (remote areas and areas located close to urban regions) shows some minor differences since the problem of recruitment to the public sector is more pronounced in remotely located rural regions than in rural regions located closer to urban areas. Hence, closeness to urban regions mitigates some of the recruitment problems. These rural areas seem to benefit from access to knowledge and better matching (Duranton and Puga 2004) in nearby urban areas. However, with regard to recruitment to the business sector, there is no statistically significant difference between the two types of rural regions. Hence, in conclusion, the overall finding is that recruitment is generally perceived as difficult regardless of location.

What are the consequences of these recruitment problems and this lack of skills? The respondents are in agreement in noting that to a large extent, recruitment problems result in a lack of skills matching and that this constitutes an obstacle to growth in a region. Seventy-eight percent of respondents in rural regions and 66% of those in nonrural regions agree that recruitment problems result in a lack of skills matching. Eighty-seven percent of respondents in rural regions and 82% of those in nonrural regions agree that difficulties with recruiting constitute an obstacle to growth. The situation in rural regions is particularly problematic, as more respondents from these areas than from nonrural municipalities state that the municipality’s geographic location is a disadvantage to recruitment efforts. Fifty-nine percent of the respondents in rural regions agree that geographic location is a disadvantage. The same figure for other municipalities is 28%, and this difference is statistically significant. It is interesting to note that this difference primarily originates from differences between the two different types of rural regions. Location is less of a problem for recruitment processes when a rural region is located close to an urban region, again suggesting that these regions benefit from accessibility to agglomeration advantages in nearby urban areas (Duranton and Puga 2004).

Our literature review provides an overview of the political measures previously employed to enhance the recruitment and retention of scarce competencies in rural regions. In the survey, respondents were asked to share their views on five of these policy measures (see Table 2). Furthermore, with an open-ended question, respondents were asked to report three policy measures at the municipal, regional or state level that they considered to be most important in facilitating the recruitment of skills shortages in a region.

**Table 2:** Policies to enhance recruitment to remote regions

	Mean	St. dv.	Difference (mean)
**To what extent do you think that the following policy measures would facilitate the recruitment of skills shortages to regions with recruitment difficulties?**			
*Support for accompanying persons in finding a job*			
Nonrural municipalities	5.429	1.756	–
Rural municipalities	6.255	1.031	0.826***
Rural and located close to urban region	6.136	0.941	–
Rural and remote location	6.360	1.114	0.224
*An incentive model for writing off student debt*			
Nonrural municipalities	4.200	1.856	–
Rural municipalities	5.150	1.929	0.950**
Rural and located close to urban region	4.778	1.927	–
Rural and remote location	5.455	1.920	0.677
*Lower social security contributions for companies in the region*			
Nonrural municipalities	4.800	1.841	–
Rural municipalities	5.068	1.860	0.268
Rural and located close to urban region	4.714	1.821	–
Rural and remote location	5.391	1.877	0.677
*Subsidized wages in the region*			
Nonrural municipalities	2.951	1.596	–
Rural municipalities	3.650	2.032	0.699*
Rural and located close to urban region	4.278	1.776	–
Rural and remote location	3.136	2.122	−1.142^#^
*Higher child allowance for families in the region*			
Nonrural municipalities	2.625	1.444	–
Rural municipalities	3.707	2.052	1.082***
Rural and located close to urban region	4.000	1.947	–
Rural and remote location	3.429	2.158	−0.571

* indicates a statistically significant difference between rural and non-rural municipalities. *p < 0.10, **p < 0.05, ***p < 0.01

# indicates a statistically significant difference between rural and located close to urban region and rural and remote locations. ^#^*p* < 0.10, ^##^*p* < 0.05, ^###^*p* < 0.01

Table 2 lists the perceived effectiveness of the five policy suggestions. The research literature reviewed in this study reveals that policy evaluations have mainly focused on financial incentives for regional recruitment and that our knowledge of nonpecuniary measures designed to stimulate recruitment is much less extensive. However, the nonpecuniary measure of providing support for accompanying persons in finding jobs received the greatest number of positive responses from both rural and nonrural regions. This was also something that was frequently addressed in response to the open-ended question. Since family structure is one of the strongest drivers in determining location decisions (Bjerke and Mellander 2017), this highlights the importance of policies that also consider family variables.

Regarding pecuniary incentives, the proposal of an incentive model for writing off student debt also received a great number of positive responses. However, if wages and pecuniary incentives for voluntary job choices and job changes (Yang 2003; Terjesen et al. 2007; Nyström and Zhetibaeva Elvung 2015) are of limited importance to applicants, this policy may be less effective. Furthermore, it may be advantageous to target recruitment to individuals who focus on aspects other than pecuniary incentives. In a field experiment, Ashraf et al. (2020) tested whether recruited individuals hired from a group focused on career opportunities perform better than those hired from a group focused on access to social benefits. The authors found individuals hired from the career opportunities group to be more talented and equally prosocial and that those hired from the career opportunities group subsequently performed better on the job, hence resulting in a better job match.

Neither subsidized salaries nor increased child allowances were perceived to facilitate recruitment to any great extent (low levels on the Likert scale). This finding is interesting, as it is in line with previous evaluations showing that pecuniary measures targeted at the firm level seem to have limited employment effects.

The results show statistically significant differences between the respondents from rural municipalities and those from nonrural municipalities for all policy measures, except in the case of lower social security contributions. Hence, respondents in rural regions view policies that can help relieve regional recruitment and matching problems more positively. However, there are no statistically significant differences in the perceptions of individuals in rural and remote regions and of those in regions located closer to urban areas.

An interesting question concerns whether there is any relationship between recruitment problems and the perceived effectiveness of different policy measures. To investigate any potential relationship, the two measures of the severity of recruitment problems (a municipality’s public activities and businesses) were correlated against perceptions of the effectiveness of policy measures. Regarding recruitment to municipality public activities, there is a positive and statistically significant correlation with the perception of an incentive structure for writing off student debt and of support for accompanying persons in finding a job (correlations of 0.269 and 0.379, respectively). There is also a statistically significant correlation between respondents who perceive it to be difficult for municipality businesses to recruit and to better support accompanying persons in finding a job (correlation of 0.331). Finally, the substantial variation found across regions, especially with respect to views on effective policy, is worth noting. Possible explanations are that industrial diversity or differences in political governance influence the perceived role of policy rather than location. This is clearly an issue that may be interesting to explore in future research.

In the following, a summary of the respondents’ thoughts on the open-ended question regarding which policies they consider to be most important in facilitating the recruitment of needed skills in a given region is provided.^7^ The suggestions is categorized into the following areas: infrastructure, education, taxes and other financial incentives and other policy measures.

### Infrastructure

Increased investment in infrastructure and communications, opportunities for teleworking with improved technology, internet connections and broadband; attractive homes with associated services; increased opportunities to build in attractive locations (sea and coastal locations).

### Education and training

Improved status for industry and craft professions; increased investment in vocational education and apprenticeships; more opportunities for employees in companies to participate in vocational training courses; more flexible training initiatives and easier paths to establishing locally adapted education; facilitation of language education/practice linked to education.

### Taxes and other financial incentives

Reduced tax burdens on rural housing; reduced tax burdens on transport in rural areas, e.g., fuel/taxes for passenger cars; government grants for municipalities to help increase the salaries of teachers and nurses; government-subsidized loans for the new construction of villas for rent in locations where there is a gap between the second-hand market and new construction.

### Other policy measures

Relocation services; tandem recruitment and relocation services attractive leisure activities.

In sum, several of these suggestions highlight a demand for a more flexible education system, which can be adapted to the regional demand for labour. The comments also highlight that incentive models must be adapted to local conditions. For instance, rules and regulations may need to be adapted to local conditions to increase the capacity to build in attractive locations. However, an interesting final reflection highlighted by one of the respondents is that it is also important to work with attitudes and communicate the positive aspects of labour markets in rural regions. The respondent felt that what the politicians communicate are high unemployment rates. While there are jobs in rural areas, they are not visible because businesses do not recruit as they once did.

## Conclusions and suggestions for further research

The successful recruitment of scarce competencies is important for sustainable regional development in rural regions. This paper studies the perceived difficulties of recruiting scarce competencies in rural regions and compares these to the same perceptions of nonrural regions in Sweden. Furthermore, the role of policy in facilitating and enhancing recruitment to and better skills matching in rural regions is discussed.

The results show that recruitment is perceived to be difficult in all regions and that the difficulty of recruiting the right skills results in a lack of skills matching and constitutes an obstacle to growth. Given that much policy attention has been given to recruitment to public sector occupations to remedy a lack of, for example, nurses and other professionals in the health care sector (Jones et al. 2019), it is interesting to observe that the difficulties are somewhat more pronounced in the business sector. For rural municipalities located close to urban areas, accessibility to labour and skills seems to mitigate some of the disadvantages of recruitment to rural regions.

The findings suggest a need to find new means for the government and local policymakers to support recruitment to both the public and business sectors. To do so, municipality officials from the business sector highlight the need for flexible policies that can be adapted to the regional demand for labour. This concerns, for example, educational efforts but also rules and regulations that could enhance the attractiveness of a region. Even though the suggestion of a pecuniary incentive model to write off student debt for those who relocate to rural regions received a very positive response, many municipalities would prefer to advance nonpecuniary policy measures, such as relocation support for accompanying persons and tandem recruitment. However, as indicated by the literature review, our knowledge of such nonpecuniary policy initiatives is still limited, highlighting a clearly interesting and important avenue for future research. In addition, recruitment based primarily on pecuniary incentives may not be very successful and may result in poor matching. Finally, the substantial variation observed across regions with respect to views on effective policy is worth noting. It may be that industrial diversity or differences in political governance influence the perceived role of policy. This is clearly an issue that may be interesting to explore in future research.

The fact that respondents in both rural and nonrural regions have similar views on which policies they primarily think would work is an interesting finding. Support for accompanying persons and written off student debt are the policies deemed most effective. However, there are some differences in perceptions of the power of these policies, where respondents from rural regions view their implementation more positively. In this case, it is of less importance if a rural municipality is remote or located close to urban areas. There is potential for policies targeted towards rural regions to create tension between regions, which may hinder the implementation of such policies. However, in this case, there is a unified view of which policies are perceived to be effective in supporting recruitment to remote regions, which may enhance implementation.

In the introduction, an example of a firm that adopted innovative recruitment strategies for a remote region in Canada is provided. How did this recruitment process in Nova Scotia evolve? When the recruiters stopped counting and selected three families, they had received more than 3000 inquiries (CBC News 2016b).

A member of the Walkins family, one of the three families recruited to Whycocomagh, said the following:

So here, we actually get to become part of a community. We’ll get to know the people. We’ll get to work with them, and we’re really excited about that. (CBC News 2016b),

Success with recruitment to rural areas requires creativity and innovativeness from employers and policymakers at both the government and local levels. However, this is a challenging task, particularly since the measures involved need to be resource efficient and effective.

## Figures and Tables

**Fig. 1:**
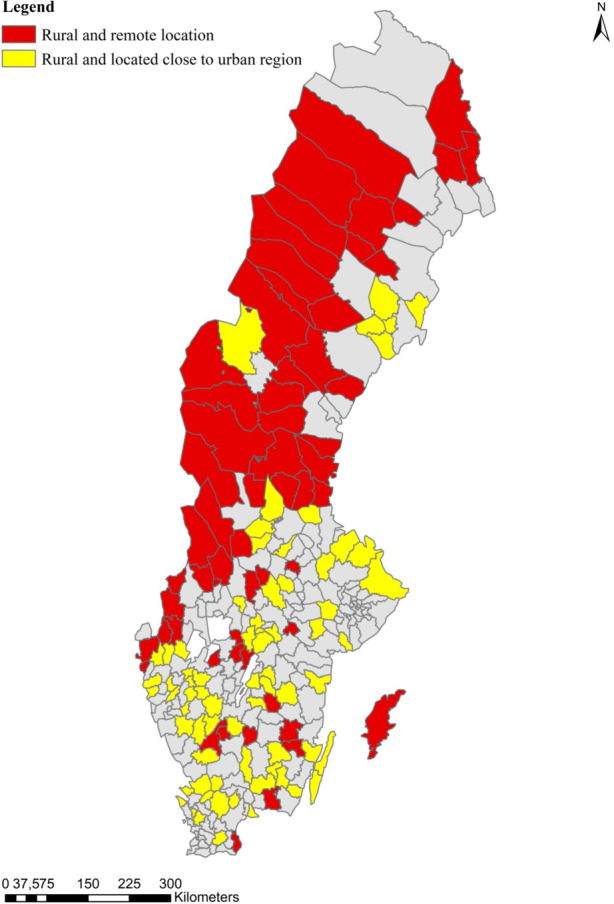
Definition of rural regions

## Footnotes

1 The delimitation of policies relevant for enhancing recruitment to rural regions is not trivial. A broad perspective on this matter may cover several regional policy areas. Nevertheless, the criterion for being included in this policy survey was that the policy was either specifically targeted towards recruitment problems or had specific implications for recruitment problems. This implies, for instance, that regional policies targeted towards companies such as incubators are not included in the discussion.

2 The complete distribution of responses is available from the author upon request.

3 The individuals’ positions and roles imply that they are municipality officials and hence are not politically elected.

4 Existing surveys related to perceived skills shortages tend to use a stratified process to select companies. This implies that the number of respondents from small regions is too low to publish findings at a very disaggregated regional level. Due to our targeting of municipalities’ officials, one should be aware of that the presented results will reflect the officials’ knowledge and perceptions of regional recruitment conditions.

5 See Swedish Agency for Growth Policy Analysis (2014) for a complete list of which municipalities are defined as rural municipalities. Fig. 1 in the Appendix provides a map indicating the two types of rural municipalities.

6 This difference is statistically significant at the 10-percent level. The difference primarily originates from nonrural regions, which perceive it to be slightly more difficult to recruit to the business sector than to the public sector. In rural regions, it is equally difficult (approximately five on the Likert scale with no statistically significant difference) to recruit to the public sector and to the business sector.

7 Here, one may also include proposals mentioning perceptions of the five policies. Hence, in the summary below, we exclude the suggestions already targeted by the quantitative question included in the survey.
